# Exome Sequencing of Consanguineous Pashtun Families With Familial Epilepsy Reveals Causative and Candidate Variants in *TSEN54*, *MOCS2*, and *OPHN1*

**DOI:** 10.1111/cge.14627

**Published:** 2024-10-14

**Authors:** Afrasiab Khan, Anees Muhammad, Hidayat Ullah, Hina Ambreen, Abeed Ullah, Patrick May, Holger Lerche, Tobias B. Haack, Shoaib ur Rehman, Josua Kegele

**Affiliations:** ^1^ Department of Biotechnology University of Science and Technology Bannu Bannu Pakistan; ^2^ Department of Molecular Biology & Genetics Institute of Basic Medical Sciences, Khyber Medical University Peshawar Pakistan; ^3^ Luxembourg Centre for Systems Biomedicine University of Luxembourg Esch‐sur‐Alzette Luxembourg; ^4^ Department of Neurology and Epileptology Hertie Institute for Clinical Brain Research, University of Tuebingen Tuebingen Germany; ^5^ Institute of Medical Genetics and Applied Genomics University of Tuebingen Tuebingen Germany

**Keywords:** epilepsy genetics, *MOCS2*, molybdenum cofactor deficiency B, neonatal epilepsy, *OPHN1*, Pakistan, Pashtun, pontocerebellar hypoplasia type 2A, *TSEN54*

## Abstract

Next‐generation sequencing is advancing in low‐ and middle‐income countries, but accessibility remains limited. In Pakistan, many members of the Pashtun population practice familial marriage and maintain distinct socio‐cultural traditions, isolating them from other ethnic groups. As a result, they may harbor genetic variants that could unveil new gene‐disease associations. To investigate the genetic basis of epilepsy in the Pashtun community we recently established a collaboration between Bannu University and the University of Tuebingen. Here we report our first results of exome sequencing of four families with presumed monogenetic epilepsy and Mendelian inheritance pattern. In Family #201, we identified distinct disease‐causing variants. One had a homozygous pathogenic missense variant in *TSEN54* (c.919G > T, p.(Ala307Ser)), linked to Pontocerebellar Hypoplasia Type 2A. The second individual had a homozygous class IV missense variant in *MOCS2* (c.226G > A, p.(Gly76Arg)) which is associated with Molybdenum cofactor deficiency. In family EP02, one affected individual carried a heterozygous class III variant in *OPHN1* (c.1490G > A, p.(Arg497Gln)), related to syndromic X‐linked intellectual disability with epilepsy. Our small study demonstrates the promise of next‐generation sequencing in genetic epilepsies among the Pashtun population. Diagnostic next‐generation sequencing should be established in Pakistan as soon as possible, and if not feasible, genetic research projects may pioneer this path.

## Graphical abstract

The Pashtun population is prone to genetic diseases due to common practice of familial marriage and socio‐cultural isolation. Through exome sequencing of four selected families, we identified causative and candidate variants in *TSEN54*, *MOCS2*, and *OPHN1*.

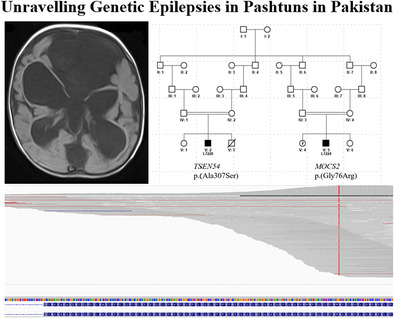

## 1 Introduction

Genetic testing is an evolving field in many low‐ and middle‐income countries (LMIC) [1]. In Pakistan, however, the implementation of public health genomics is hampered by a number of existing problems, including cultural and health‐system issues [2]. This contrasts with data showing a high burden of genetic diseases in Pakistan: for example, Yaqoob et al. reported consanguineous marriage rates of 46%–67% in Pakistan [3]. By using next‐generation sequencing and other genetic diagnostics within the Pashtun population, many new gene‐disease associations have been revealed already. For instance, Riazuddin et al. identified 30 new candidate genes associated with intellectual disability by testing 121 consanguineous families [4].

Our research team from the Department of Biomolecular Sciences at the University of Science and Technology Bannu (USTB) observed a high rate of epilepsy within consanguineous Pashtun families. Consistent with these observations, recent reports indicate that the prevalence of cousin marriages among parents of patients with epilepsy stands at 37% for first degree cousins and 16% for second degree cousins [5], suggesting a significant genetic influence on disease mechanisms within this community. To unravel the genetic background of epilepsies in the Pashtun population we established an equal research collaboration between the USTB and the University of Tuebingen.

Here we present our first results of our pilot study where we performed exome sequencing (ES) of six patients from four families with presumed monogenetic epilepsy.

## 2 Materials and Methods

The study was performed according to local regulations and was approved by the Ethical Review Board of the USTB (ref. no. USTB/Biotech/1143). The legal guardians of the participants provided written informed consent. All study‐related procedures adhered to the ethical standards of the Declaration of Helsinki and its later amendments. Patients visited the Neurology Department of the Lady Reading Hospital in Peshawar, Pakistan, where neurologists clinically diagnosed epilepsy via patient history, physical examination, electroencephalography (EEG) and magnetic resonance imaging (MRI). Affected members of Family #201 were additionally examined by J.K. and H.L. during a visit to Pakistan in 11/2021.

**FIGURE 1:**
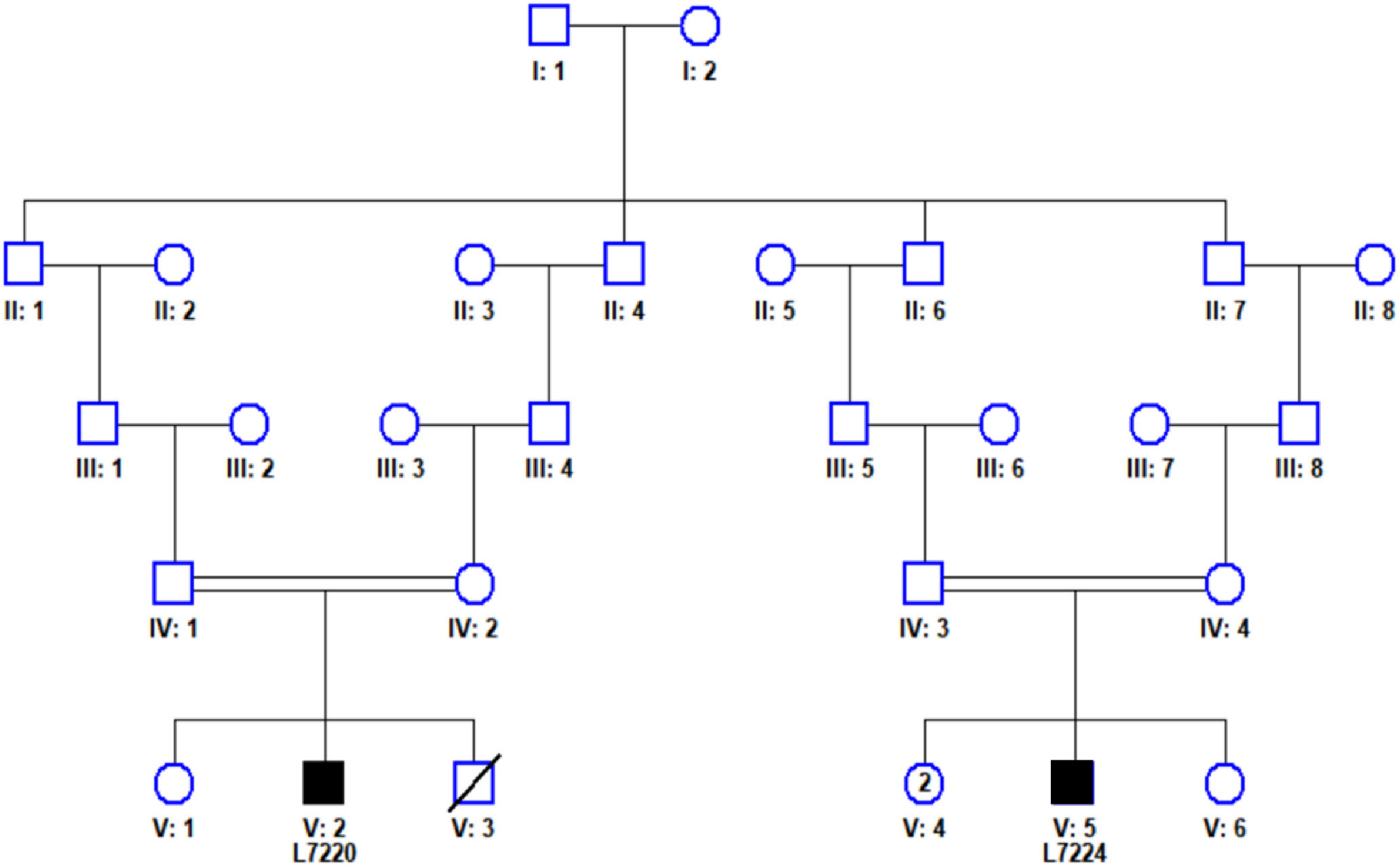
Pedigree of Family #201. Black squares represent affected individuals.

Clinical data were obtained directly from patients, medical charts, or the responsible physician. EDTA samples from available family members were sent to the Hertie Institute for Clinical Brain Research, University of Tuebingen, where DNA was extracted using standard methods. The pedigree in Figure 1 was created via HaploPainter V1.043.

ES was conducted at the Institute of Medical Genetics and Applied Genomics Tuebingen as described previously [6]. The sequence data were analyzed using the megSAP pipeline (https://github.com/imgag/megSAP), which includes CNV calling, and the data were aligned to the GRCh38 reference genome. Causative variants were validated via Sanger sequencing. The genetic variants were interpreted following ACMG standards [7].

## 3 Results

Family #201: Two male third degree cousins (L7270 and L7274, Figure 1) reported with neonatal‐onset epilepsy and severe developmental delay. The parents of each affected individual were second degree cousins (see Figure 1) suggesting an autosomal recessive disorder in both individuals, potentially with the same homozygous genetic defect.

**FIGURE 2:**
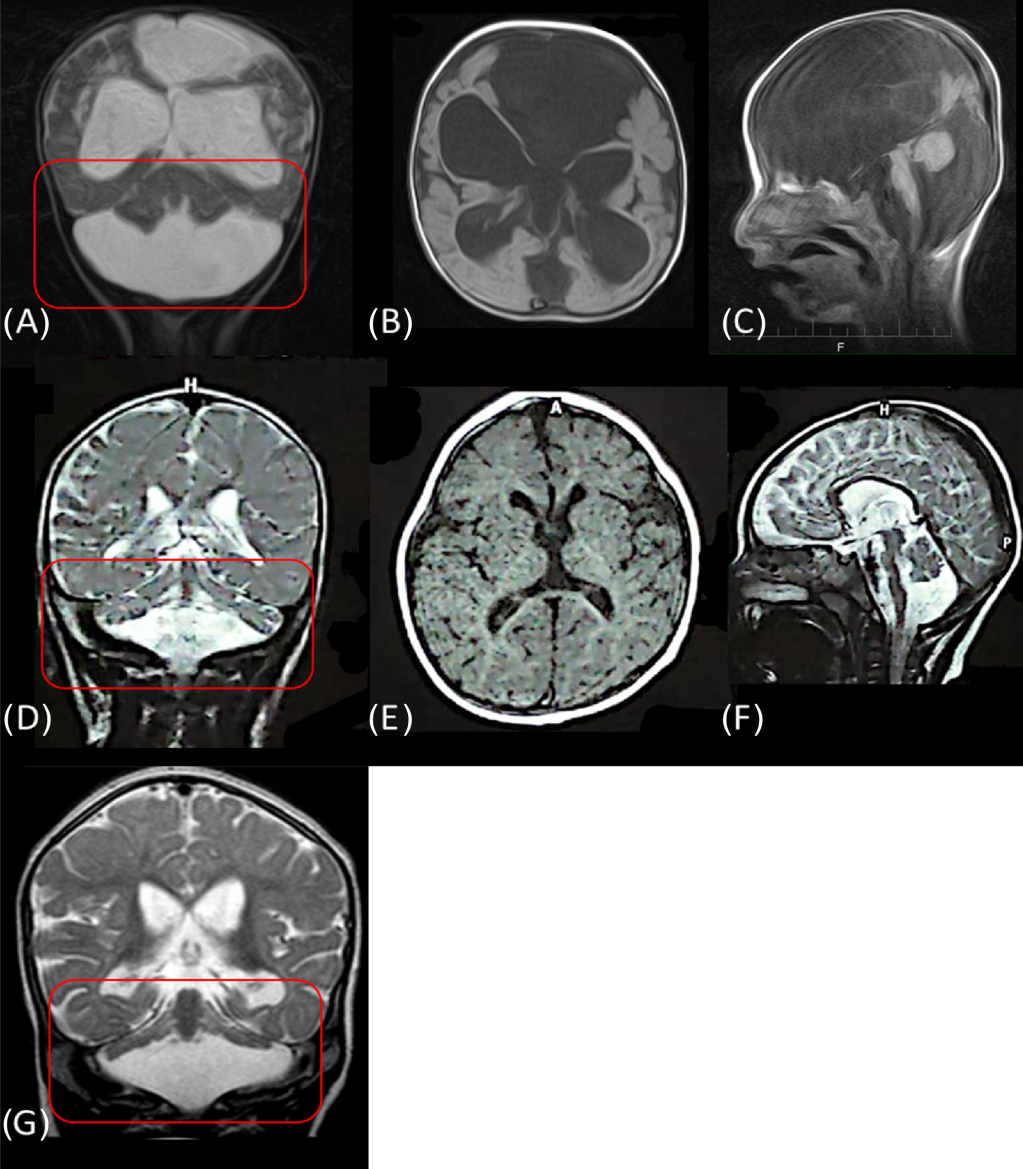
MRI findings of Family #201 compared with the literature of patients with PCH2A. A–C: MRI of L7274, carrying the class IV *MOCS2* variant (c.226G > A) in homozygous state showing a complex supra‐ and infratentorial brain malformation with enlarged cerebrospinal fluid space; D–F: MRI from patient L7270 with a confirmed pathogenic homozygous *TSEN54* variant (c.919G > T) with flattened cerebellar hemispheres (circled red), also known as dragon sign. G: dragon sign of a patient with the same homozygous variant as patient L7270 [8].

Individual L7270, a 2‐year‐old boy of 10 kg, had his first seizure with 15 days of age. Pregnancy and delivery were normal but development was delayed achieving head control not before the age of 1 year. He showed a complex oculomotor paresis and strabismus. In contrast to the second individual (L7274), he was able to control his gaze and fix his mother's eyes. He had partial head control, mild non‐directed spontaneous movements of all extremities, but was unable to sit. Muscle tone was diminished. MRI showed flattened cerebellar hemispheres (“Dragon sign,” Figure 2) [8].

ES revealed a pathogenic homozygous missense variant in *TSEN54* (c.919G > A, p.(Ala307Ser)) in individual L7270 which is the most common variant causing autosomal recessive pontocerebellar hypoplasia type 2a (PCH2A; OMIM #277470). The parents were tested for the variant and are heterozygous carriers. *TSEN54* encodes subunits of tRNA splicing endonuclease complex involved in RNA processing. It is highly expressed in neurons of the pons, olivary nuclei, and cerebellar dentate during the second trimester of fetal brain development. It has been hypothesized that loss‐of‐function variants may impair the ability to cleave pre‐tRNAs by the endonuclease complex, affecting the development of these structures [9]. However, the exact disease mechanism, including the development of epilepsy is not well understood. Figure 2 shows the MRI of our patient compared to typical MRI changes of patients with pontocerebellar hypoplasia.

Individual L7274 a 16‐months‐old boy weighing 8 kg, had neonatal‐onset multifocal myoclonic seizures and focal to bilateral tonic–clonic seizures. Pregnancy and delivery were normal, no CNS trauma or infection occurred before the onset of seizures. He experienced developmental regression at the age of 3 months, losing had control. Physical examination showed a hypotrophic child without visual fixation, absent pupillary light reflex, severe flaccid tetraparesis without spontaneous movements. The right side exhibited increased tendon reflexes, whereas both, Troemner and Babinski signs were normal. The patient was taking baclofen and clobazam which could mask an enhanced muscle tone in the physical examination. He also had mild dysmorphic features (macrocephalus, frontal bossing, low‐set eyes) MRI showed a severe complex brain malformation (Figure 2).

He was tested negative for the *TSEN54* variant even though the phenotype and MRI features are somewhat similar, although much more severe, exceeding the phenotype of PCH2A. Interestingly, ES revealed a homozygous class IV variant in *MOCS2* (c.226G > A, p.(Gly76Arg)). *MOCS2* encodes molybdenum cofactor, which is essential for the activities of sulfite oxidase, xanthine dehydrogenase, aldehyde oxidase, and mitochondrial amidoxime reducing component, all of which play critical roles in metabolic processes [10, 11]. Loss‐of‐function variants in *MOCS2* are linked with autosomal recessive molybdenum cofactor deficiency B (MOCODB), which leads to clinical manifestations such as poor feeding, pharmacoresistant epilepsy, severe developmental delay, intracerebral cysts, and complex brain malformations while typically maintaining the brain stem intact. Most affected patients die in childhood (OMIM #252160). The homozygous variant found in our patient is located at the last base of exon 4. Computational predictions suggest a moderate impact according to MaxEntScan and a high confidence in alteration of splicing according to SpliceAI (0.91 score). The sequence is highly conserved and rare in the general population, with a frequency of 0.00041 in gnomAD when heterozygous. Homozygous variants are not present in gnomAD. Based on our literature review, functional analyses have not been performed for this variant, but two independent patients with MOCODB carrying the identical homozygous variant in *MOCS2* have been reported, exhibiting a phenotype consistent with the clinical presentation of our patient [10]. Laboratory abnormalities supporting the diagnosis of MOCODB (e.g., hypouricemia, increased urinary xanthine, hypoxanthine, S‐sulfocysteine, and taurine) cannot be confirmed as the patient passed away. The consanguineous non‐affected parents have not been tested for variant carrier status.

Family EP02: Individual L7228, son of consanguineous (cousin) parents, suffers from a neonatal‐onset generalized epilepsy with generalized motor seizures and global developmental delay. EEG showed generalized epileptiform discharges. His sister also suffered from unclassified epilepsy and died of unknown causes, while his three brothers are unaffected. There were no signs or history of acquired epilepsy (including normal cerebral CT‐scan).

ES revealed a hemizygous variant in *OPHN1* (c.1490G > A, p.(Arg497Gln)). *OPHN1* (OMIM*300127) encodes oligophrenin‐1, which is involved in neuronal cell migration and maturation and plasticity of excitatory synapses [12]. Pathogenic variants in *OPHN1* cause X‐linked intellectual disability with ataxia, seizures, strabismus, but also cerebellar hypoplasia and ventriculomegaly (#OMIM 300486), neither of which have been seen on the CT‐scan in our patient. However, patients with causative *OPHN1* variants and normal cerebral imaging have also been reported [13]. In silico tools (phyloP and CADD) predict a deleterious effect for this variant. The sequence is highly conserved and rare, with an allele frequency of 0.00003 in gnomAD when hemizygous. Conflicting interpretations exist on ClinVar (Class II/III). The parents have not been tested for the variant but the carrier status of the unaffected mother and wildtype status of non‐affected male siblings would support the hypothesis of pathogenicity. There is no evidence of X‐linked inheritance from the pedigree.

We did not identify candidate copy number variations in any of the patients. The results are summarized in Table 1.

**TABLE 1:** Summary of Results.

L‐Nr	Pak‐Nr	Gene	RefSeq	cDNA	Amino‐acid alteration	Zygosity
L7270	EP201	*TSEN*54	NM_207346	c.919G > T	p.Ala307Ser	Homozygous
L7274	EP201	*MOCS*2	NM_004531	c.226G > A	p.Gly76Arg	Homozygous
L7228	EP02 VII:1	*OPHN*1	NM_002547	c.1490G > A	p.Arg497Gln	Hemizygous
L7222	EP02 VI:4	n/a**	—	—	—	—
L7233	EP18 IV:2	n/a	—	—	—	—
L7237	EP14 VI:1	n/a	—	—	—	—

L‐Nr	GnomAD AF	CADD score	Classification	ClinVar ID	Phenotype
L7270	0.00089 (het)	27.5	P	2120	PCH2A (details see Section 3)
L7274	0.00001 (het)	34.0/SpliceAI 0.91	LP* (PM2, PP3, PP4, PP5)	419958	MOCODB (details see Section 3)
L7228	0.00001	24.3	VUS (PM2, PP3, BP6)	2300429	Male, generalized epilepsy, onset at birth, combined developmental delay, EEG: generalized epileptiform discharges, CT‐scan normal (details see Section 3)
L7222	—	—	—		Female, otherwise like L7228
L7233	—	—	—		Female, generalized and focal epilepsy, onset at age of 4 years, seizure type unknown, no comorbidities (normal development), EEG: focal and generalized epileptiform discharges, no imaging
L7237	—	—	—		Female, focal epilepsy, onset at birth, several seizures per month, moderate delayed global development, EEG: frontal non‐lateralized epileptiform discharges, CT‐scan normal

*Note*: Classified as pathogenic or likely pathogenic according to the American College of Medical Genetics and Genomics (ACMG) guidelines.

Abbreviations: *for justification we refer to the Section 4; **n/a, not applicable because no candidate variant identified; AF, allele frequency; L‐Nr, laboratory number (Hertie Institute Tuebingen); LP, likely pathogenic; MOCODB, molybdenum cofactor deficiency B; P, pathogenic; Pak‐Nr, laboratory number at the University of Science and Technology Bannu; PCH2a, Pontocerebellar Hypoplasia Type 2a; VUS, variant of unknown significance.

## 4 Discussion

In our pilot study, we identified a pathogenic homozygous *TSEN54* variant in a patient with early‐onset epilepsy. The results highlight the importance of ES since—reviewing established epilepsy panels [14, 15, 16, 17]—panel sequencing might not have detected the variant, even though neonatal seizures have been described in PCH2A [18, 19]. These cases suggest considering *TSEN54* in neonatal‐onset epilepsy, particularly if phenotypic information is incomplete, which may be the case not only in resource‐poor countries. In our PCH2A patient, detailed phenotypic data, including MRI, facilitated genotype–phenotype correlation and confirmed the diagnosis.

The homozygous *MOCS2* splice site variant's classification ranges from Class III to V on ClinVar. We found descriptions of two unrelated individuals who were diagnosed with MOCODB and carried the same variant as our patient [10]. Both showed severe phenotypes, similar to our patient, including early‐onset seizures, spastic tetraparesis, and blindness. One patient's MRI revealed ventricular dilatation resulting from global cerebral atrophy, while the other patient's MRI were not detailed. Considering the evidence for pathogenicity mentioned in the results and the reported clinical features of individuals carrying the same variant, we classified the variant as Class IV. Laboratory tests may have helped to confirm the diagnosis, but sadly, the patient passed away before specific urinary test could be performed.

The heterozygous variant in *OPHN1* in patient EP02 VII:1 is difficult to interpret. Our patient suffers from seizures and combined developmental delay which includes intellectual disability consistent with the *OPHN1* phenotype. Additional phenotyping including MRI, IQ‐testing, and segregation analysis are necessary the pathogenicity of this variant.

Conclusion: Our findings support considering *TSEN54* in neonatal‐onset epilepsy and favoring next generation sequencing over panel diagnostics, even in resource‐poor settings. Detailed phenotyping, including brain MRI and genetic testing for all affected family members has helped to avoid misinterpretation in Family #201 and should always be pursued to enhance diagnostic accuracy. Our preliminary study has led to establishing procedures for large‐scale studies on familial epilepsies in the Pashtun population within our German–Pakistanian collaboration. To improve genetic testing access in LMICs, we propose to establish close collaborations between established genetic institutes in high‐income countries and researchers and clinical centers in LMICs. These collaborations would involve exchange and training of clinicians, bioinformaticians and researchers to ultimately reduce the gap and disparities in genomic diagnosis, contributing to democratizing genetic testing in LMICs [20]. Diagnostic genetic testing should be established in Pakistan at soonest and if not (yet) possible, research projects may pioneer this path.

## Author Contributions

Conception and design of the study: H.L., S.R., J.K.; Acquisition of data: A.K., A.M., H.U., H.A., A.U., H.L., S.R., J.K.; Analysis of data: A.K., A.M., P.M., H.L., T.H., S.R., J.K.; Drafting the text and preparing the figure and tables: A.K., A.M., S.R., J.K. All authors commented on previous versions of the manuscript.

## Conflicts of Interest

The authors declare no conflicts of interest.

### Peer Review

The peer review history for this article is available at https://www.webofscience.com/api/gateway/wos/peer‐review/10.1111/cge.14627.

## Data Availability

The data that support the findings of this study are available from the corresponding author upon reasonable request.
